# Influence of Graphene Type and Content on Friction and Wear of Silicon Carbide/Graphene Nanocomposites in Aqueous Environment

**DOI:** 10.3390/ma15217755

**Published:** 2022-11-03

**Authors:** Bernadette Schlüter, Christian Schröder, Wenli Zhang, Rolf Mülhaupt, Ulrich Degenhardt, Richard Sedlák, Ján Dusza, Katalin Balázsi, Csaba Balázsi, Andreas Kailer

**Affiliations:** 1 Fraunhofer Institute for Mechanics of Materials IWM, Woehlerstr. 11, 79108 Freiburg, Germany; 2 Freiburg Materials Research Center (FMF), Institute for Macromolecular Chemistry, University of Freiburg, Stefan-Meier-Str. 21, 79104 Freiburg, Germany; 3 QSIL Ingenieurkeramik GmbH, Rauenstein, Gewerbepark 11, 96528 Frankenblick, Germany; 4 Division of Ceramic and Non-Metallic Systems, Institute of Materials Research, Slovak Academy of Sciences, Watsonova 47, 040 01 Košice, Slovakia; 5 Centre for Energy Research, Institute for Technical Physics and Materials Science, Hungarian Academy of Sciences, 1121 Konkoly-Thege Miklos Str. 29-33, H-1525 Budapest, Hungary

**Keywords:** silicon carbide, graphene nanocomposite, graphene coating, spark plasma sintering, friction, wear

## Abstract

Two different types of graphene materials were used as functional nanofillers for the mechanical and tribological improvement of silicon carbide/graphene nanocomposites. On the one hand is thermally reduced graphite oxide (TRGO) reduced at three different temperatures, and on the other hand is graphene made of three different organic precursors, which were directly coated on silicon carbide (SiC) platelets (GSiC). Additionally, benchmark materials were also used as carbon fillers. The SiC/graphene nanocomposites with 2 wt% filler content were manufactured by pressureless sintering (PLS). Some composites were produced with higher graphene contents of 4% and 8% and sintered by spark plasma sintering (SPS). Microstructural analyses were conducted using scanning electron microscopy (SEM) and transmission electron microscopy (TEM). Underwater lubrication, the SP sintered TRGO and GSiC materials with high graphene content have shown the most promising tribological performance. Furthermore, the reduced size of the homogeneously distributed nanoparticles promotes the formation of surface states, which improve the friction and wear properties.

## 1. Introduction

There is a strongly growing demand for highly wear-resistant and reliable ceramic materials that may be widely used in industrial applications and energy production. Reliability and efficiency of these components need to be improved by using high-performance ceramics with superior tribological and good mechanical properties. The main aim of this work is to develop novel, highly efficient materials on the basis of graphene-containing silicon carbide (SiC) nanocomposites and to demonstrate their suitability for technical applications such as slide bearings and face seals in aqueous media. SiC is an advanced ceramic material which is characterized by good mechanical and chemical properties as well as a superior resistance against wear [1,2]. For this reason, SiC ceramics are suitable for slide bearings and face seals, which are exposed to high tribological loads during operation. Nevertheless, there is a need to improve the friction and wear properties of these ceramic components and thereby enhance the reliability and the application range. The microstructural processes and wear mechanisms occurring in SiC under tribological loading in water lubrication have been extensively investigated by Presser et al. [2]. Nevertheless, further investigations are necessary to predict the technical reliability, especially when the ceramic material is modified by carbon fillers. In this respect, various research projects have been carried out; however, mainly silicon nitride and alumina ceramics with carbonaceous fillers such as CNT [3], graphene nanoplatelets (GNP) [4] and reduced graphite oxide (rGO) [5] were used. As summarized in recent reviews [6,7] the mechanical strength of silicon carbide ceramics may be only moderately increased by graphene and graphene-like platelets, whereas their fracture toughness is enhanced significantly. The strengthening mechanisms were considered to be crack deflection, crack bridging, tearing-open areas, relative sliding between graphene layers and crack branching. Some fewer publications also deal with tribological aspects. Here, however, the focus has been partly on other ceramic materials such as Si_3_N_4_ [8,9] and boron nitride [10] ceramics in combination with graphene. These studies showed reduced friction and enhanced wear resistance; however, they were conducted under dry sliding, without any direct reference to real technical applications. Apart from the work on SiC/graphene ceramics, which focus on mechanical properties [11,12], fracture toughness [13] and various sintering methods [14], a few publications also discuss tribological properties under dry sliding conditions [15] or under lubrication with isooctane [16]. Though their wear resistance increases due to the formation of a surface-protecting layers, the friction behavior was not necessarily improved under dry conditions in comparison to unfilled SiC ceramics [15,17,18].

As reported by Zhang et al. [19], mechanochemically functionalized multilayer graphenes (MGs) affect the tribological behavior when used as fillers in SiC/MG nanocomposites. Under water-lubricated conditions, a noticeable reduction in friction and wear was achieved in comparison to standard SiC ceramics. It was found that MGs promote the formation of a wear protective surface layer.

The research work presented in this paper follows on from the studies of Zhang et al. [19] investigating graphene-containing SiC nanocomposites. Here, the focus is set on both thermally reduced graphite oxide (TRGO) and graphene coated SiC (GSiC) as filler materials. Advantageously, TRGO is well-dispersible [20] and can thus be distributed homogeneously in a ceramic matrix. In turn, GSiC can be produced quickly and easily and, thus, cost-effectively, which is a manufacturing advantage over established graphene production processes. It is also possible to use biobased raw materials for the production; thus, GSiC takes eco-friendly production and sustainability into account [18]. In addition, both TRGO and GSiC can be manufactured with spark plasma sintering (SPS), a process which allows improved material properties to be achieved in shorter process times compared to conventional sintering processes [21]. Complementary to the work of Zhang et al. [19], the following study also examines the influence of different filler contents on the mechanical and tribological properties. The results are furthermore compared with the friction and wear behaviors of SiC ceramics containing commercially available carbon fillers.

## 2. Experimental Section

### 2.1. Synthesis of Thermally Reduced Graphite Oxide (TRGO)

Graphite oxide (GO) was prepared by oxidizing graphite via the Hummers method [22]. Graphite (60 g, KFL99.5 graphite, AMG Graphite, Hauzenberg, Germany) and NaNO_3_ (30 g) were suspended overnight in H_2_SO_4_ (95%, 1.4 L). At 0 °C, the KMnO_4_ (180 g) was added in portions within 2 h. The suspension was then stirred for 2 more hours at room temperature. The dispersion was poured slowly on ice water (1.4 L), and H_2_O_2_ (5%, 200 mL) was added. The resulting mixture was filtered in a buechner funnel. The solid filter cake was rinsed several times with deionized H_2_O (4 × 5 L) and concentrated HNO_3_ (67%, 4 × 400 mL). The resulting sulphate and manganese impurities in the GO were largely removed by cross-flow filtration [23]. After drying, approx. 80 g GO was obtained. The reaction product was dried for 7 days in a vacuum oven at 40 °C under reduced pressure and pulverized by a cryogenic mill (Retsch GmbH, Haan, Germany) (90 s, 25 s^−1^) with nitrogen cooling. After milling, the drying procedure was repeated. The dry powder was reduced in a tube furnace at 600 °C, 800 °C and 1000 °C under nitrogen atmosphere (Typ RSR 120/750/11, Nabertherm, Lilienenthal, Germany) to yield a low-density powder. Accordingly, the produced thermally reduced graphite oxides are referred to as TRGO-600, TRGO-800 and TRGO-1000. To integrate the particles homogeneously into the ceramic matrix, TRGO dispersions (pH 10, 1 g/L) were produced in water. For this purpose, the dispersion was dispersed once in the high-pressure homogenizer (Panda NS1001L 2K, GEA Niro Soavi, Parma, Italy) at 1000 bar after an ultrasonic bath pretreatment (2 × 15 min) [20].

### 2.2. Synthesis of Graphene Coated SiC (GSiC)

For the preparation of GSiC, three different precursors were used: dopamine (GSiC-D) [24], furfuryl alcohol (GSiC-F) [18] and glucose (GSiC-G) [25,26].

#### 2.2.1. GSiC-D

Graphene-coated silicon carbide from dopamine (GSiC-D) was first prepared by dispersing SiC (18 g, PRC XF 13, 13 m^2^/g, d_90_ = 2 µm, QSIL Ingenieurkeramik, Frankenblick, Germany) in tris(hydroxymethyl)–aminomethane solution (80 mL; 100 mmol/L in H_2_O) using an ultrasonic bath (15 min). Then the solution of dopamine hydrochloride (2.00 g) was added to the tris(hydroxymethyl)–aminomethane solution (20 mL; 100 mmol/L in H_2_O). After stirring the reaction mixture for 72 h the water was removed by freeze-drying. The powder was mortared and carbonated in the tube furnace (Typ RSR 120/750/11, Nabertherm, Lilienenthal, Germany) in a nitrogen atmosphere. The material was heated to a temperature of 150 °C at a rate of 5 K/min, maintained for 21 h, and then increased to 1000 °C at 5 K/min and thermolyzed for 6 h.

#### 2.2.2. GSiC-F

Graphene-coated silicon carbide from furfuryl alcohol (GSiC-F) was produced by dispersing SiC (152.55 g, PRC XF 13, 13 m^2^/g, d_90_ = 2 µm, QSIL Ingenieurkeramik Frankenblick, Germany) in water (200 mL). This was followed by the addition of furfuryl alcohol (15 mL). Then, after 10 min stirring, p-TsOH (0.33 g in water 10 mL) was added. After 20 min of stirring, the reaction mixture was heated up to 80 °C and held at this temperature for 1 h. After 6 h, the mixture was stirred at 100 °C until the powder was dry. After crushing the powder with a mortar, it was carbonized in the tube furnace (Typ RSR 120/750/11, Nabertherm, Lilienenthal, Germany) in a nitrogen atmosphere. The material was heated up to a temperature of 150 °C at a rate of 5 K/min, maintained for 21 h, and then increased to 800 °C at 5 K/min and thermolyzed for 6 h.

#### 2.2.3. GSiC-G

Graphene-coated silicon carbide from glucose (GSiC-G) was produced by dispersing SiC (20 g, PRC XF 13, 13 m^2^/g, d_90_ = 2 µm, QSIL Ingenieurkeramik Frankenblick, Germany) in water (90 mL) by ultrasonic bath (2 × 15 min). Then the solution of D(+)-glucose (2.00 g in 10 mL water) was added. After predispersion by using an ultrasonic bath (2 × 15 min), the reaction mixture was completely dispersed under ice cooling via an ultrasonic lance. After freeze-drying, the powder was mortared and carbonated in the tube furnace (Typ RSR 120/750/11, Nabertherm, Lilienenthal, Germany) in a nitrogen atmosphere. The material was heated up to a temperature of 150 °C at a rate of 5 K/min, maintained for 21 h, then increased to 800 °C at 5 K/min and thermolyzed for 6 h.

### 2.3. Benchmark Materials

Four different carbon fillers were chosen as commercially available benchmark materials: Graphite (KFL99.5 graphite AMG Mining), carbon black (CB, Derussol N25/L, Orion Engineered Carbons, Cologne, Germany) and two types of benchmark graphene nanoplatelets (BG): BG-grade C (GnP^®^ Grade C–750) and BG-grade M (GnP^®^ Grade M–15) both delivered by XG sciences.

### 2.4. Powder Analysis of TRGO, GSiC and Benchmark Materials

Elemental analysis (carbon, hydrogen, nitrogen and sulfur content) was performed by using a vario MICRO cube (Elementar Analysensysteme GmbH, Langenselbold, Germany). The specific surface area was measured by the adsorption of nitrogen according to Brunauer–Emmett–Teller (BET) theory by using a Sorptomatic 1990 (POROTEC GmbH, Hofheim, Germany). The graphene content on top of the coated SiC-particles was measured by thermogravimetric analysis (10 K/min, 50–750 °C, air atmosphere, STA 409, Netzsch GmbH, Selb, Germany). The mass loss was determined as the filler content in comparison to pure SiC [21]. Scanning electron microscopy (SEM) images of the powder of benchmark materials were obtained by using a Quanta 250 FEG (FEI, Thermo Fisher Scientific, Freiburg, Germany) with an ETD detector and an accelerating voltage of 20 kV or 30 kV. The TEM images of TRGO and GSiC powder were taken with a LEO 912 Omega transmission electron microscope (TEM) (Zeiss, Oberkochen, Germany) at 120 kV acceleration voltage. High-resolution transmission electron microscopy (HRTEM) for GSiC powder was performed with a JEM-3010 from JEOL, Freising, Germany at an acceleration voltage of 300 kV.

### 2.5. Production of SiC/Graphene Nanocomposites

SiC/graphene nanocomposite slurries were prepared by blending SiC (PRC XF 13, 13 m^2^/g, d_90_ = 2 µm, QSIL Ingenieurkeramik, Frankenblick, Germany) and sintering aids (B_4_C, carbon black) with the desired amount of graphene material in a planetary ball mill (PM4, Retsch GmbH, Selb, Germany) for the duration of 4 h and a sharker mixer (TURBULA Type T2F, Willy A. Bachofen AG, Muttenz, Germany) for another 4 h. TRGO was incorporated in the form of a stable aqueous dispersion (pH 10, 1 g/L). After screening (grain size 63 μm), the sample was redried and granulated by freeze-drying. The granules (grain size < 180 μm) were then compacted to test plates (cold isostatic pressing, 2000 bar) and sintered by a conventional solid phase sintering process using sintering parameters that generally lead to highly densified microstructures for silicon carbide ceramics (sintering temperature, 2050 °C; argon atmosphere, 4 h; without pressure). Test specimens were milled from the sintered plates.

Additionally, selected formulations containing 4% TRGO-600, 4% TRGO-1000 and 4% GSiC-D were sintered via spark plasma sintering (SPS). SPS is a new technique and enables fast and energy-efficient sintering of ceramic parts. However, SPS is only possible with sufficient contents of electrically conductive fillers. This was the case for samples with 4% and 8% of graphene fillers. For this sintering process, the conductive powder (>20 Ωcm) was compacted between two highly conductive graphite punches (Ø 60 mm, 60 MPa). A current was applied to the punches (max. 10 V) until the sinter temperature 2050 °C was reached. The sample was then sintered at this pressure and temperature for 5 min in an argon atmosphere.

The density of SiC/graphene nanocomposites was measured by using Archimedes’ method in water. The four-point bending strength (σ) values of ceramic nanocomposites were determined in accordance with DIN EN 843-1 on bars with dimensions 3 mm × 4 mm × 45 mm at ambient temperature and atmosphere. The fracture toughness was measured by the Single Edge V-notch Beam (SEVNB; DIN EN ISO 23146) method. The specimens with dimensions 3 mm × 4 mm × 45 mm were ground and polished by a 15 µm diamond grinding wheel before testing. A sharp V-notch using the razor blade and diamond paste (6 µm, 3 µm and 1 µm for the final stage of notching) with total depth of between 0.8 mm and 1.2 mm was prepared. Electrical resistance measurements were performed on specimens with a dimension of 3 mm × 4 mm × 45 mm. The contact area of the specimens was coated with silver paste to assure good contact with the crocodile clip. The specific conductivity was calculated from these values.

### 2.6. Tribological Tests

Sliding tests of graphene-infiltrated SiC ceramics against commercially available SSiC face seal counter rings (outer diameter: 60.8 mm, inner diameter: 44 mm, R_a_: 0.65 µm, Hexaloy SA, Eagle Burgmann, Wolfratshausen, Germany) were performed using a standard pin-on-ring setup (TRM 1000, Dr.-Ing. Georg Wazau Mess- + Prüfsysteme GmbH, Berlin, Germany). The graphene-containing pins had a spherical tip with a radius of 5 mm and had dimensions of 10 mm × 4 mm × 4 mm in length, width and thickness. The contact geometry of a spherical tip ensures that an initial point of contact is established; thus, there is no risk of contact misalignment. The initial contact stresses were calculated according to Hertzian equations. The normal force and sliding speed were 50 N and 0.1 m/s; consequently, the initial contact pressure amounts measured up to 2.7 GPa. A drawback of the use of a spherical tip radius is that, due to wear of the pins, the contact area continuously increases during the sliding test, which leads to a continuous decrease in the contact pressure. In order to avoid hydrodynamic influence, which would affect the comparability of the results, the test duration was restricted to 4 h. It was therefore ensured that the contact pressures were at least 10 MPa throughout the whole test. The tests were conducted under water-lubricated conditions at room temperature and repeated twice. As reference material combination, SSiC pins without graphene (SSiC#137) were tested against SSiC face seal counter rings under the same conditions.

The pins’ surfaces were investigated subsequently after the tribological tests via digital microscopy and SEM (ZeissSupra 40VP, Carl Zeiss AG, Oberkochen, Germany) to determine the wear volumes and wear mechanisms. In the SEM analyses, a low electric voltage of 3 kV and a SE detector were used to resolve surface changes due to wear with high contrast. For selected samples, atomic force microscopy (AFM) was carried out to detect tribologically induced layer formation on top of the surfaces (Dimension V, Bruker, Billerica, MA, USA).

## 3. Results

### 3.1. Synthesis and Characterization of TRGO and GSiC

**Figure 1:**
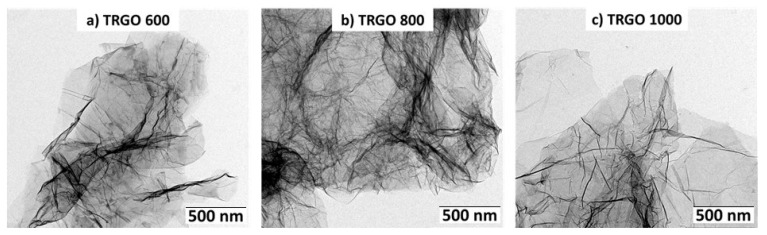
TEM images of TRGO after the dispersing process.

As it is apparent from Table 1, the oxygen content (functionality) of the resulted wrinkled TRGO platelets decreases from 15.4 wt% for TRGO-600 down to 5.1 wt% for TRGO-1000, while the specific surface area increases up to 515 m²/g by rising reduction temperature. Through the dispersion process in water using the high-pressure homogenizer, the graphene stacks are additionally separated, and the specific surface area is further increased. TEM images of dispersed TRGO are shown in Figure 1.

**Figure 2:**
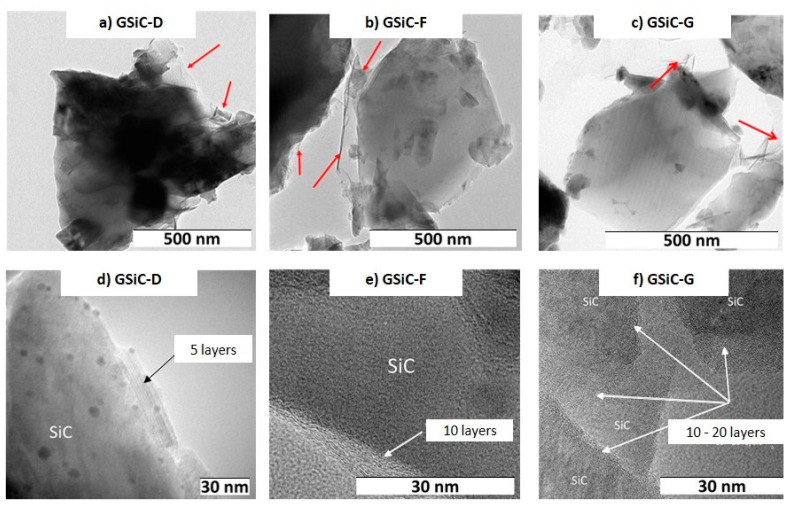
TEM images of GSiC materials: (**a**) GSiC-D (5.2% graphene), (**b**) GSiC-F (4.4% graphene), (**c**) GSiC-G (5.2% graphene) and HRTEM images of GSiC materials: (**d**) GSiC-D (5.2% graphene), (**e**) GSiC-F (4.4% graphene), (**f**) GSiC-G (5.2% graphene). The arrows show graphene with few layers.

As shown in the TEM images in Figure 2, the SiC platelets were coated by graphene material (see arrows). The HRTEM images of the different GSiC powders show the number of layers that are also related with the specific surface area of these materials. The fewer the layers, the higher the specific surface area. For GSiC samples with 14% graphene, specific surface areas of 70 m²/g for GSiC-G, 100 m²/g for GSiC-F and 110 m²/g for GSiC-D were reached.

### 3.2. Characterization of Benchmark Materials

**Figure 3:**
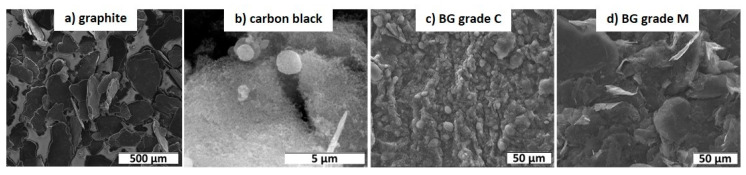
SEM images of the benchmark materials.

The morphology and particle sizes of the four different benchmark materials are shown in Figure 3. While graphite is quite large, the other fillers are smaller. Furthermore, the particle morphologies are different, as graphite and BG-grade M have a platelet-like structure, but carbon black and BG-grade C are spherical. Additionally, graphite consists of almost pure carbon, while the other three benchmark materials contain between 6 wt% and 10 wt% oxygen (Table 1). It was found that BG-grade C has by far the biggest specific surface area in comparison to the other materials.

### 3.3. Preparation and Characterization of SiC/Graphene Nanocomposites

#### 3.3.1. Density, Mechanical and Electrical Properties

Table 2 provides an overview of the manufactured composites and their density, electrical conductivity and mechanical properties. At higher filler content, slightly lower sinter densities were obtained. The SPS-sintered samples show higher sinter densities, although they have higher filler content. Especially for the SPS-sintered samples (SiC + 4% TRGO-1000 and 4% GSiC-D) and the 2% BG-grade C and 8% GSiC-F–containing sample, the conductivity increases significantly. Generally, the conductivity is higher at higher filler content for all filler types. Furthermore, the reduction temperature of TRGO has an influence on the electrical conductivity, as with higher temperature the conductivity is increased due to the higher carbon content of TRGO and therefore fewer defects. The four-point bending strength and the fracture toughness decrease with increasing graphene content. This does not apply to the SPS-sintered specimens, which have significantly better values for the GSiC containing material.

#### 3.3.2. Tribological Properties

**Figure 4:**
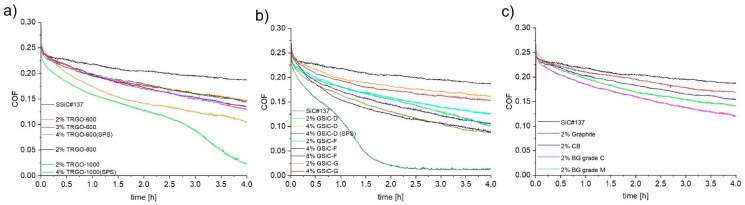
Friction behaviors of different SiC nanocomposite pins sliding against SSiC in aqueous environment at room temperature with 50 N and 0.1 m/s; (**a**) TRGO with different reduction temperatures; (**b**) GSiC materials with different precursors; (**c**) benchmark materials.

Figure 4 displays the average coefficient of friction (COF) of different material combinations using SiC/graphene nanocomposite pins sliding against SSiC counter rings in an aqueous environment at room temperature. For comparison, the black curve in Figure 4 depicts the friction development of the reference combination of SSiC pins sliding against SSiC rings. Figure 4a shows the COF of the tribo-pairs with ceramics containing TRGO pins. While all materials show a friction decrease with test duration, the lowest values were obtained for the TRGO-containing pins. For the samples filled with 2% TRGO, there is no clear influence of the reduction temperature during synthesis of the TRGO (600 °C, 800 °C, 1000 °C) on the friction behavior. In contrast, the different friction curves correlate with the TRGO amount in the composite as higher contents result in lower COFs. Accordingly, the lowest COFs were in the range of approx. 0.03 for the material containing 4% TRGO-1000(SPS) and 0.11 for that containing SiC + 4% TRGO-600(SPS), whereas the COF of the reference materials containing no TRGO ended with a COF of 0.19. Furthermore, for TRGO, the reduction temperature affects the friction behavior of the SPS-sintered samples: The higher the reduction temperature, the stronger the decrease in the COF is. This is due to the lower oxygen content and higher specific surface area in TRGO-1000, which leads to finer grain size and better distribution.

**Figure 5:**
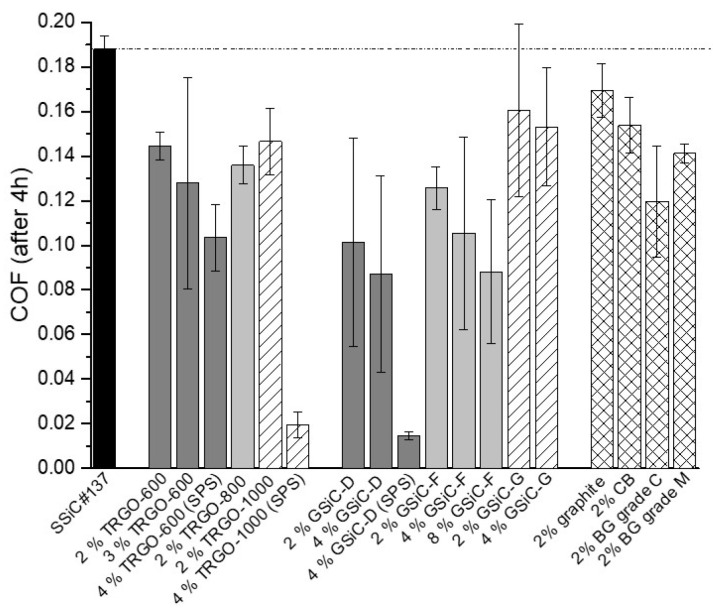
COF after 4 h of the different graphene-containing SiC nanocomposite pins sliding against SiC rings in aqueous environment.

Figure 4b shows the friction courses of the SiC pins containing GSiC materials against SSiC rings. Again, friction continuously decreases to values that are significantly lower than the COF of the reference system. As with TRGO-filled ceramics, the COF is lower at higher filler content for all selected precursors. The COFs of the best materials reach final values of approx. 0.09 after 4 h. Generally, rather high friction values were maintained with GSiC G materials that were synthesized with glucose as the precursor. The difference of the friction behavior between nanocomposites using furfuryl alcohol (GSiC F) and dopamine (GSiC D) as precursors is not significant. By far the lowest friction values were observed for SiC + 4% GSiC-D (sintered by SPS), which reached a COF of approx. 0.01 after 3.5 h. In contrast, the identical powder composition sintered normally only reached friction values of 0.09. Figure 4c illustrates the friction values of benchmark materials. In accordance with the friction behaviors of the other systems with graphene-containing SiC samples, the COFs also reached slightly lower values than the reference combination. The composite with the coarsest filler (graphite) resulted in the highest COF of 0.17, followed by CB (0.15) and BG-grade M (0.14). The lowest COF of 0.12 was reached with the filler BG-grade C. A comparative overview of the frictional behavior is depicted in Figure 5, in which the coefficient of friction after 4 h test duration in the form of a bar chart with error bars. This diagram clearly shows that the friction values of the graphene-containing materials are strongly reduced and ultralow friction is partly reached.

**Figure 6:**
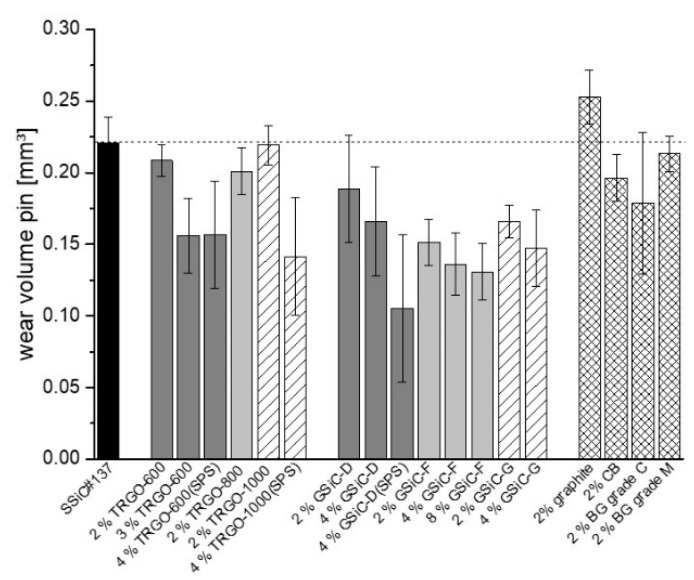
Wear of the different graphene-containing SiC nanocomposite pins after sliding against SiC rings for 4 h in aqueous environment.

The wear results of the pins are shown in Figure 6. This overview shows that the wear of all tested materials is roughly in the same order of magnitude. Compared to the reference materials, a wear reduction in the range of 20 to 50% may be reached by using graphene-containing materials. The only exception is SiC + 2% graphite, which showed slightly higher wear than the reference SSiC#137 material. Generally, increasing the graphene content, regardless of whether TRGO or GSiC, lead to a reduction in wear. It should be emphasized that SPS pins generated noticeably lower wear.

**Figure 7:**
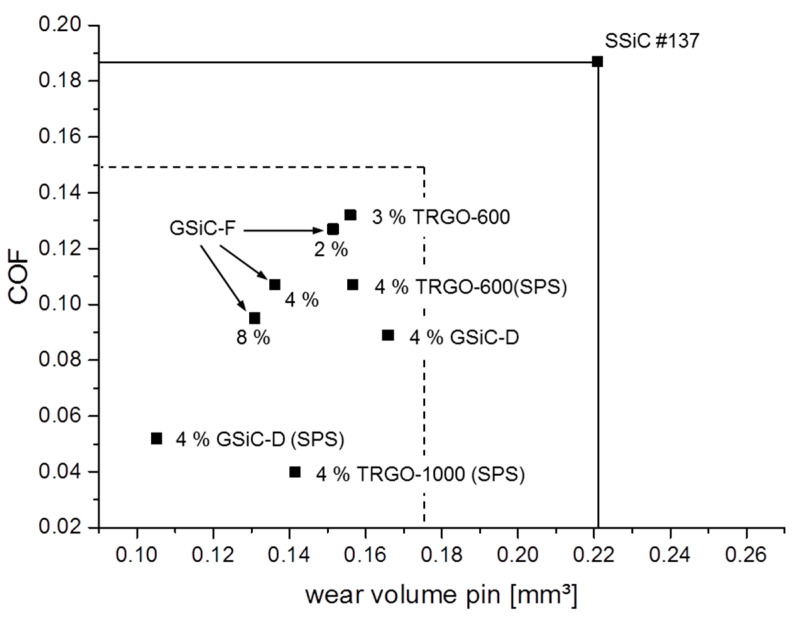
COF vs. wear volume of selected pin materials (20% better than reference material) measured after 4 h test duration.

In order to summarize the best results of the pin-on-ring tests, the COFs measured after 4 h are plotted in relation to the respective wear volumes for selected tribosystems in Figure 7. The two solid lines refer to COF and wear volume of the system with reference material without filler. In relation to these values, the dashed lines refer to COF and wear volumes, which are reduced by 20%. Therefore, the drawn data points mark those graphene-containing materials, which resulted in improved friction and wear by at least 20%.

### 3.4. Surface Analysis after Tribological Testing

**Figure 8:**
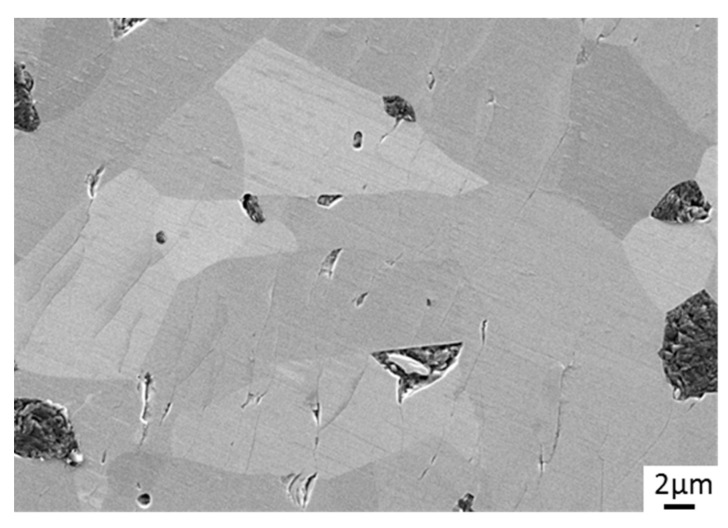
Microscopic image (SEM) of the SSiC#137 reference pin after tribological testing.

The worn surface of the reference material SSiC#137 is shown in Figure 8. Under water lubrication, the contact surfaces of SiC materials generally become very smooth due to tribochemical interaction with water. There is a typical microstructure for SiC ceramics that is relatively coarse-grained (ca. 20 µm grain size) and contains some inclusions and small pores. In addition to small striations and grooves that indicate the sliding direction under a very mild wear regime, several surface cracks that are oriented perpendicular to the sliding direction indicate initial surface degradation by solid–solid interaction between the sliding surfaces.

**Figure 9:**
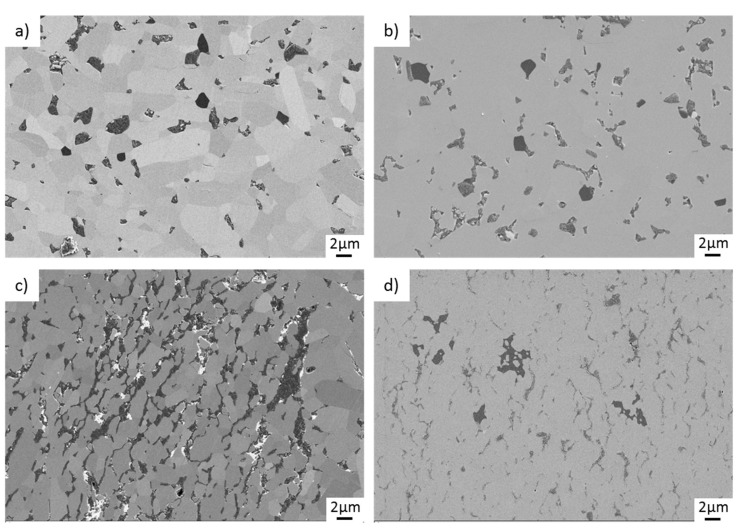
Microscopic images (SEM) of pin surfaces after tribological testing. (**a**) SiC + 2% TRGO-600, (**b**) SiC + 2% TRGO-800, (**c**) SiC + 4% TRGO-600(SPS), (**d**) SiC + 4% TRGO-1000(SPS).

In contrast, there is no microcracking on the surfaces of the TRGO-containing pins after sliding (Figure 9). Sinter additives as well as TRGO are visible within the fine-grained (up to 5 µm) SiC matrix. The TRGOs within the SPS-sintered variants (Figure 9c,d) accumulate at the grain boundaries and are characterized by a preferred orientation.

**Figure 10:**
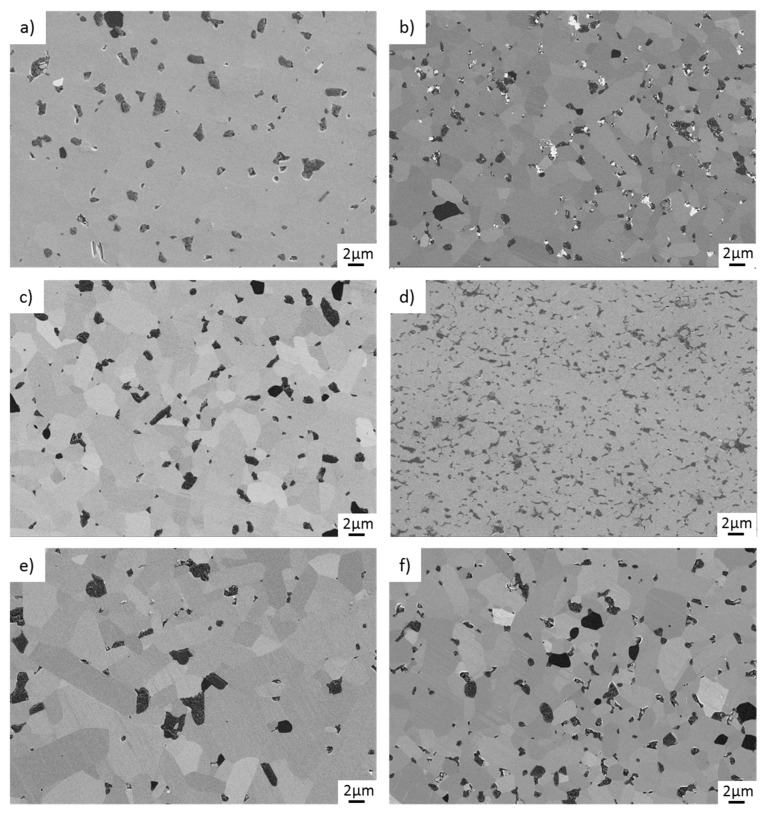
Microscopic images (SEM) of pin surfaces after tribological testing. (**a**) SiC + 2% GSiC-F, (**b**) SiC + 4% GSiC-F, (**c**) SiC + 2% GSiC-D, (**d**) SiC + 4% GSiC-D (SPS), (**e**) SiC + 2% GSiC-G, (**f**) SiC + 4% GSiC-G.

Figure 10 shows the worn surfaces of GSiC-containing pins after sliding. The same material types are arranged in the same row. They differ in the filler content; the left figure shows the 2% graphene-containing nanocomposite, and the right figure, the 4% graphene-containing microstructure. None of the surfaces show microcracking, tribochemically induced surface modifications or manufacturing defects such as pores. Rather, the surface is smoothed by the mild tribochemical wear in the presence of water so that only very fine striations are visible in the sliding direction. However, it should be clear that very thin surface layers or tribochemical reaction products cannot be detected by a conventional SE detector. As it can be seen, the microstructures of all variants become finer with increasing GSiC content. The grain size amounts to between 1 and 10 µm. It should be emphasized that SiC + 4% GSiC-D (SPS) is characterized by intergranular accumulation of carbon at the grain boundaries.

**Figure 11:**
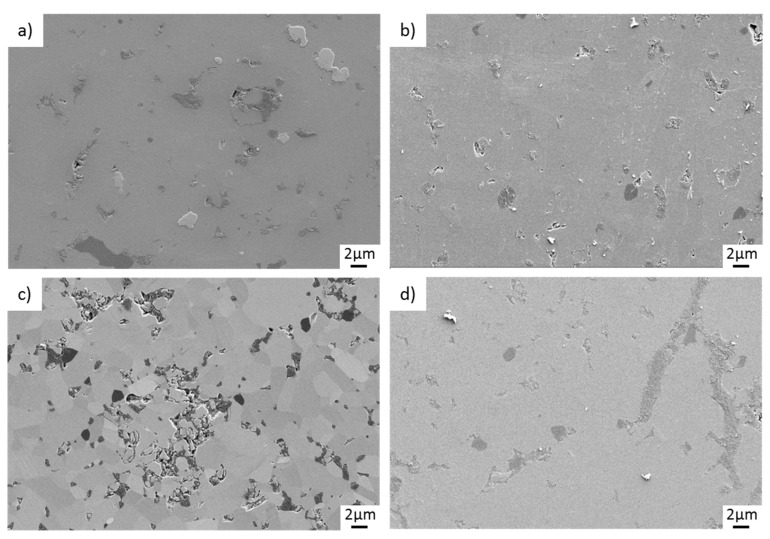
Microscopic images (SEM) of pin surfaces after tribological testing. (**a**) SiC + 2% graphite, (**b**) SiC + 2% CB, (**c**) SiC + 2% BG-grade C, (**d**) SiC + 2% BG-grade M.

Finally, Figure 11 illustrates the SiC pins containing benchmark materials after tribological loading. Especially Figure 11a,c show microstructures, which consist of large filler particles that are heterogeneously distributed and shaped. In contrast, the sizes and dispositions of carbon particles shown in Figure 11b,d are comparatively finer. For these two variants, micropores are visible. Just like the surfaces of TRGO- and GSiC-containing SiC pins, no microcracking can be identified.

**Figure 12:**
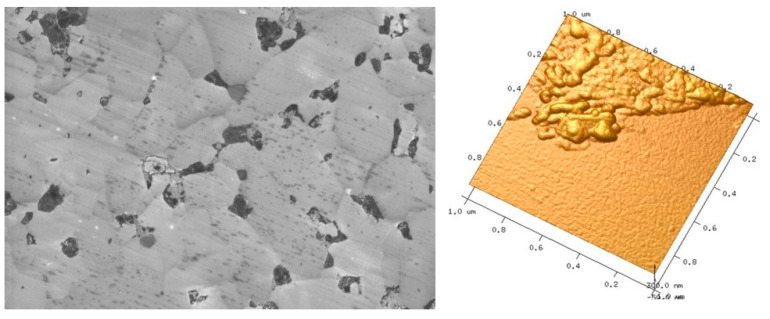
In-lens detection (**left**) and AFM analysis (**right**) of the worn pin surface made of SiC + 4% GSiC-F.

A more surface-sensitive analysis was carried out with scanning electron microscopy using an in-lens detector and, additionally, AFM of the worn surfaces. Figure 12 shows representative results of such analyses for of the pin made of SiC + 4% GSiC-F that superficial deposits. At very high magnification, these deposits appear as inhomogenously distributed amorphous residues with sizes in the range of a few submicrons in size and less than 50 nm in height.

**Figure 13:**
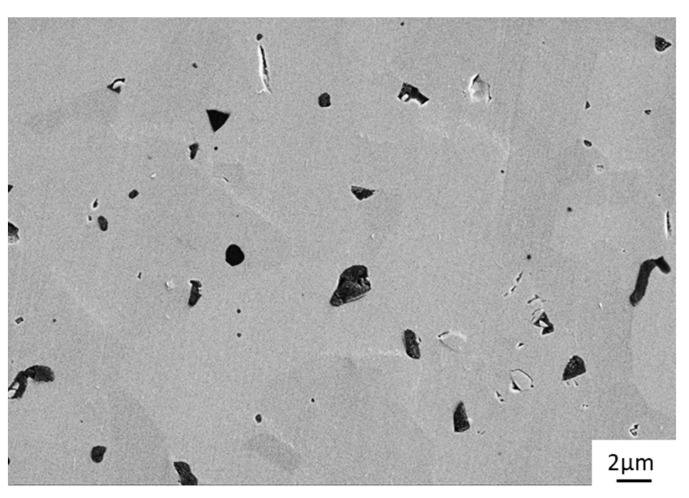
Microscopic image (SEM) of SiC ring surfaces after tribological testing against SiC + 2% GSiC-F.

A representative SEM image of a worn surface of a silicon carbide ring is shown in Figure 13. Like in all tests conducted in this study, the surface has become very smooth. Despite some pores and inclusions, consisting of boron carbide sintering aids, there are no visible irregularities or tribologically induced microcracks.

## 4. Discussion

The starting point of this research work was the analysis of the tribological behavior of SiC ceramics containing graphene in aqueous media, in light of a technical application of these innovative materials for bearings in pumps. Therefore, two different types of graphene materials were chosen: thermally reduced graphite oxide (TRGO), reduced at three different temperatures (600 °C, 800 °C and 1000 °C), and graphene made from three different organic precursors, which was directly coated on SiC platelets (GSiC-F, GSiC-G, GSiC-D). Additionally, four commercially available carbon benchmark materials were chosen as fillers.

The material synthesis of SiC-based nanocomposites is very complex, since it depends on a lot of subsequent manufacturing steps, starting with powder synthesis, mixing and processing, shaping and sintering. Our goal was to reach considerable graphene contents to improve tribological properties without losing too much mechanical strength. Concerning the mechanical properties, it is very difficult to reach sufficient mechanical strength (>300 MPa) at higher graphene contents. The problem may be at this point that the carbon fillers act as a diffusion barrier during the sintering process, thus preventing densification and formation compact and pore-free microstructures. If agglomeration cannot be completely prevented, the formation of large microstructural defects is inevitable. In light of the mechanical properties (see Table 2), it is noticeable that the best mechanical strength values were reached with the GSiC types. Here, up to 4 wt% of filler could be used without reducing strength below 400 MPa. Only at 8 wt% of filler did the strength drop to approx. 300 MPa. This indicates that with the GSiC-type nanocomposites, homogeneous microstructures with acceptable mechanical properties may be realized. Further increase in the filler content may improve the friction and wear properties, but, as shown by both the TRGO and GSiC variants, will reduce the mechanical strength.

Focusing on the microstructural aspects, in the case of GSiC materials, the precursor has to be taken into account. As shown in the results, different numbers of graphene layers can be obtained on the SiC depending on the precursor. In accordance with the observed friction and wear behavior of GSiC-D and GSiC-G, the following applies: The tribological properties are improved if SiC is coated with only few graphene layers so that the filler can be distributed finer within the matrix.

From the results obtained, meaningful correlations can be made between the size of the specific surface and the mechanical and tribological properties. The specific surface area of BG-grade C is significantly increased compared to the other benchmark materials, and a significantly improved tribological behavior can be observed. A high specific surface area of the graphene correlates with good mechanical and tribological properties, as they are distributed homogeneously in the SiC matrix, resulting in a defect-free microstructure. For normally sintered SiC ceramics containing 2% TRGO, however, no influence of the specific surface on the tribological behavior can be observed. It is assumed that here the difference in the specific surface is not large enough to significantly affect friction and wear.

In addition, the positive influence of SPS sintering leads to improved microstructural properties, which have a positive effect on mechanical and tribological performance. A necessary condition for this sintering process is the sufficiently high electrical conductivity of the ceramic green body. This can be improved by increasing the filler content and by a homogeneous distribution of the graphene in the SiC matrix.

In this respect, ceramics with already good tribological properties such as 4% GSiC-containing SiC nanocomposites provided evidence that they are predestined for this process.

In general, the results of the rotating pin on disc tribotests in water show that tribological improvements, i.e., reduction of friction and lower wear rates, can be achieved with increasing the filler content. Furthermore, the tribological experiments show that a homogeneous distribution of small graphene particles in the SiC matrix is desirable in order to guarantee favorable friction and wear properties. On the one hand, the homogeneous distribution ensures that graphene is provided reliably and uniformly on the tribologically stressed surface, which is a fundamental prerequisite for the formation of a carbon-containing tribological film. This layer, in turn, has a friction-reducing effect, as it was verified in previous experimental works [19] and atomistic simulations of graphene-covered surfaces [27]. As visible in Figure 10d, the microstructure of the material 4% GSiC-F(SPS) is significantly more fine-grained than the other materials, and the distribution of the graphene inclusions is very homogeneous. This may be a possible reason for the exceptionally low friction values, since the graphenes may be more effectively released within the contact area. On the basis of these results and theories on the wear mechanisms of SiC and carbon as described in the scientific literature, we propose the following tribological mechanisms to be of major importance:

1. Tribochemical reactions: It is well-known that, under water lubrication, SiC reacts chemically with H_2_O to form strongly hydrated silica layers on the sliding surfaces [28,29]. These silica layers act as lubricious oxides and may stabilize friction at a low level.
2. Surface passivation by graphenization: There is a strongly growing interest in liquid superlubricity, which means lubricated frictional contacts with extremely low friction coefficients (lower than 0.01) [30,31,32]. Although the similar materials or environments have not exactly been investigated, it is described that ceramic materials such as silicon carbide or silicon nitride may react with surrounding carbon, present as coatings or lubricants, and chemically interact in frictional contacts to form chemically passivating, graphene-like surface terminations. The subsequent absence of chemical interaction of these passivated sites with liquid molecules or the solid frictional counterface may strongly reduce macroscopic friction.

Our present results have shown that pure SiC ceramics actually may show low and stable friction under water lubrication. However, at high contact pressures, these low-friction conditions that completely depend on silica formation may become unstable [2]. In contrast, if it is possible to passivate the ceramic surfaces at least partially by surface graphenization, even lower friction and more stable friction conditions may be reached. This effect would explain the generally lower friction of the graphene-containing SiC nanocomposites, as observed in our work (see Figure 4 and Figure 5). Moreover, the drop of the COF to ultralow friction values that was observed in two cases with nanocomposites containing 4 wt% graphene filler (see Figure 4) may also be explained by surface graphitization.

## 5. Summary and Conclusions

In this paper, various SiC/graphene ceramic composites were examined to explore their potential as materials for face seals. Therefore, three groups of composites containing TRGO, GSiC and benchmark materials as carbon fillers were produced and tribologically tested under water-lubricated conditions. In addition to sufficiently good mechanical strength and fracture toughness, all composites—except graphite—show better friction and wear performances in an aqueous environment than the SSiC#137 reference material without graphene. Significant improvement (better than 20%) was observed for higher filled and SPS-treated samples such as all SiC + GSiC-F (2%, 4% and 8%) materials, SiC + 4% GSiC-D (also with SPS), SiC + 3% TRGO-600, SiC + 4% TRGO-1000 (SPS) and SiC + 4% TRGO-600 (SPS). The results show that, besides the increase in the filler content, a homogeneous distribution of small graphene particles that are well-bonded to the ceramic matrix is necessary to achieve excellent results.

With respect to previous and recent publications on wear mechanisms, we propose as a combined tribological mechanism: (1) The tribochemical formation of strongly hydrated silica acting as lubricious oxides, and (2) the release graphene during the wear process leading to partial surface graphenization and therefore chemical passivation.

This material approach will therefore be very interesting in the future to develop tribological systems that may even enable stable liquid superlubricity, i.e., friction coefficients lower than 0.1.

## Figures and Tables

**Table 1:** Properties of the TRGO materials.

Sample	C ^(a)^	O ^(b)^	H ^(a)^	N ^(a)^	Specific Surface Area ^(c)^	TGA-Mass Loss ^(d)^	Structure
	(wt%)	(wt%)	(wt%)	(wt%)	(m^2^/g)	(wt%)	
TRGO-600	78.3	15.4	0.3	0.3	403	36.5	wrinkled platelet
TRGO-800	84.2	11.1	0.5	-	417	30.9	wrinkled platelet
TRGO-1000	89.2	5.1	0.4	0.4	515	22.5	wrinkled platelet
BG-grade C	88.0	10.7	1.1	0.5	794	21.4	amorphous, spherical
BG-grade M	90.9	5.9	0.9	-	88	10.1	platelet
graphite	99.6	0.7	0.2	0.2	3	1.9	platelet
carbon black	90.1	8.1	1.8	-	44	21.4	spherical

^(a)^ Determined by elemental analysis. ^(b)^ Determined by energy dispersive X-ray spectroscopy (EDS). ^(c)^ N_2_-Physisorption measurement for surface determination according to Brunauer, Emmett and Teller (BET). ^(d)^ Nitrogen, 50–1500 °C, 10 K/min.

**Table 2:** Density, electrical conductivity and mechanical properties of SiC/graphene nanocomposites.

	Sample	Amount of Filler	Theoretical Density	Density after Sintering	Relative Density	Electrical Conductivity	4-Point Bending Strength	SEVNB ^(a)^ Fracture Toughness
		(wt%)	(g/cm^3^)	(g/cm^3^)	(%)	(S∙cm^−1^)	(MPa)	(MPa∙m^1/2^)
**SiC**	Reference	0	3.174	3.17	99.9	3.4 ∙ 10^−8^	505 ± 65	3.09 ± 0.19
**TRGO**	TRGO-600	2	3.149	3.10	98.4	3.8 ∙ 10^−7^	350 ± 53	n.a.
	TRGO-600	3	3.128	3.03	96.9	1.25 ∙ 10^−1^	387 ± 67	n.a.
	TRGO-600 (SPS)	4	3.116	3.08	96.10	6.04	241 ± 24	n.a.
	TRGO-800	2	3.149	3.05	96.11	1.8 ∙ 10^−2^	371 ± 17	n.a.
	TRGO-1000	2	3.149	3.04	96.12	6.8 ∙ 10^−2^	377 ± 28	2.60 ± 0.13
	TRGO-1000 (SPS)	4	3.116	3.07	96.13	18.18	332 ± 49	3.44 ± 0.07
**GSiC**	GSiC-D-2%	2	3.149	3.14	96.14	1.9 ∙ 10^−8^	485 ± 117	3.52 ± 0.43
	GSiC-D-4%	4	3.126	3.04	96.15	6.6 ∙ 10^−2^	428 ± 78	3.02 ± 0.25
	GSiC-D-4% (SPS)	4	3.116	3.09	96.16	3.2 ∙ 10^−1^	540 ± 91	3.96 ± 0.06
	GSiC-F-2%	2	3.149	3.15	96.17	3.7 ∙ 10^−8^	507 ± 24	3.02 ± 0.21
	GSiC-F-4%	4	3.126	3.08	96.18	1.2 ∙ 10^−6^	434 ± 27	3.72 ± 0.25
	GSiC-F-8%	8	3.082	2.92	96.19	2.3 ∙ 10^0^	292 ± 47	2.84 ± 0.19
	GSiC-G-2%	2	3.149	3.12	96.20	1.9 ∙ 10^−8^	401 ± 23	3.70 ± 0.46
	GSiC-G-4%	4	3.126	3.07	96.21	1.2 ∙ 10^−3^	383 ± 80	2.99 ± 0.15
**Benchmark System**	Grade C	2	3.149	3.09	96.22	1.1 ∙ 10^−7^	415 ± 44	2.74 ± 0.17
	Grade M	2	3.149	2.93	96.23	6.8 ∙ 10^−1^	138 ± 6	n.a.
	Graphite	2	3.149	3.07	96.24	6.0 ∙ 10^−7^	126 ± 8	2.25 ± 0.35
	Carbon Black	2	3.149	3.15	96.25	9.1 ∙ 10^−7^	461 ± 63	n.a.

^(a)^ Fracture toughness was measured by the Single Edge V-Notched Beam (SEVNB) method. n.a. means: not available.
